# Macrophage Activation Syndrome in Adult-Onset Still’s Disease: Challenges in Early Detection and Management

**DOI:** 10.7759/cureus.80633

**Published:** 2025-03-15

**Authors:** Anil Regmi, Cecelia Hale, Nang Lin, Kevin K Kinduell, Sampath K Ethiraj

**Affiliations:** 1 Internal Medicine, Parkview Health, Fort Wayne, USA

**Keywords:** adult onset stills disease, evanescent skin rash, high ferritin, macrophage activation syndrome (mas), on off fever

## Abstract

Adult-onset Still's disease (AOSD) is an immunological disorder that manifests with fever, evanescent rash, leukocytosis, and arthralgia. One of the most severe complications of AOSD is macrophage activation syndrome, a life-threatening complication characterized by hyperactivation of the immune system and multiorgan dysfunction. This case report describes a 24-year-old female who developed macrophage activation syndrome in the setting of AOSD. Initially, she developed heterogeneous and nonspecific symptoms of fever, pharyngitis, rash, lymphadenopathy, and migratory arthralgia after a trip to Mexico. Thus, the diagnosis and appropriate treatment were delayed. Although her symptoms were temporarily relieved with oral steroids, she worsened clinically. She developed a widespread rash, persistent fever, a very high ferritin level (29,972 ng/mL), and elevated liver enzymes with mild hepatosplenomegaly, raising concern for macrophage activation syndrome. After ruling out infections, she was diagnosed with AOSD, and treatment with intravenous steroids was started, resulting in clinical improvement. Macrophage activation syndrome is a rare but fatal complication. Early recognition, particularly with elevated ferritin, liver dysfunction, and thrombocytopenia, is very important. Early intervention with glucocorticoids and biologics like anakinra is crucial for improving outcomes. The patient is in remission with ongoing follow-up with rheumatology.

## Introduction

Adult-onset Still’s disease (AOSD) is a rare inflammatory disorder characterized by a salmon-colored maculopapular rash, quotidian fever, leukocytosis, and arthralgia [1]. AOSD is a relatively uncommon disorder and was first described in children by George Still in 1896 AD [2]. While Still’s disease is a term that describes systemic juvenile idiopathic arthritis, AOSD represents the same manifestation in patients above 16 years of age [3]. The etiology of AOSD is unknown, and it has been suggested that both genetic factors and various infectious agents contribute to its development [1]. The clinical course of AOSD is characterized by three primary patterns: monophasic, intermittent, and chronic [4]. Treatment of AOSD depends on the severity of the disease (i.e., mild, moderate, or severe) and includes NSAIDs alone, anakinra, glucocorticoids, or disease-modifying antirheumatic drugs (DMARDs) [1].

AOSD can result in several complications, including macrophage activation syndrome (MAS), amyloidosis, pulmonary arterial hypertension, disseminated intravascular coagulation, thrombotic thrombocytopenic purpura, and diffuse alveolar hemorrhage [1]. Among these, one of the most severe complications is MAS, often called a “cytokine storm.” MAS occurs in a very small proportion of patients with AOSD. It is an immunological disorder in which the immune system becomes overactive, resulting in multisystem end-organ dysfunction that can be life-threatening [5]. MAS occurs in the setting of various rheumatological diseases, most typically in patients with Still’s disease. However, it can also occur in patients with systemic lupus erythematosus and Kawasaki disease [5]. This case report describes MAS as a complication in an adult patient with Still’s disease.

## Case presentation

**Figure 1:**
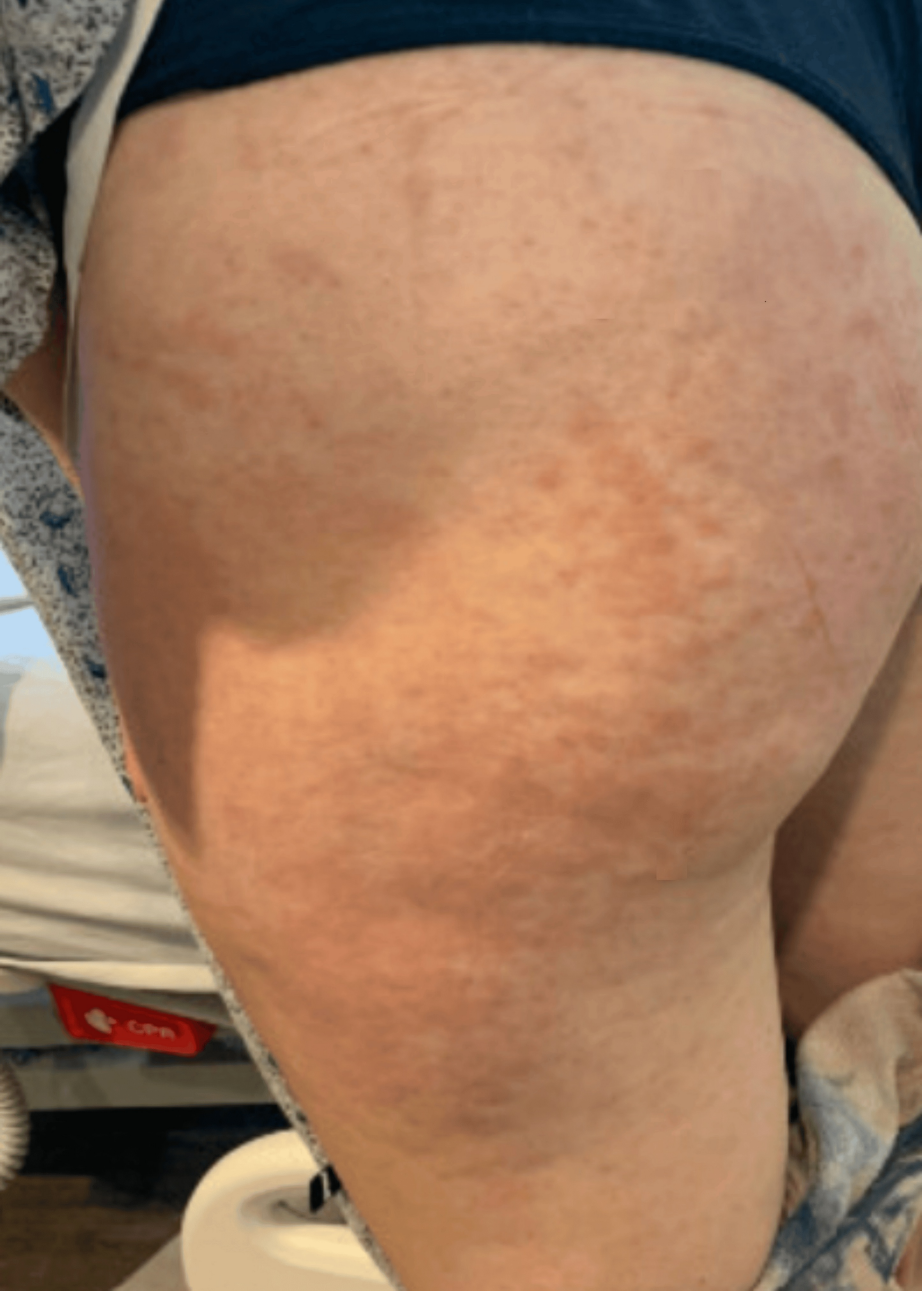
Salmon-colored maculopapular rash in buttocks and lateral thigh

**Figure 2:**
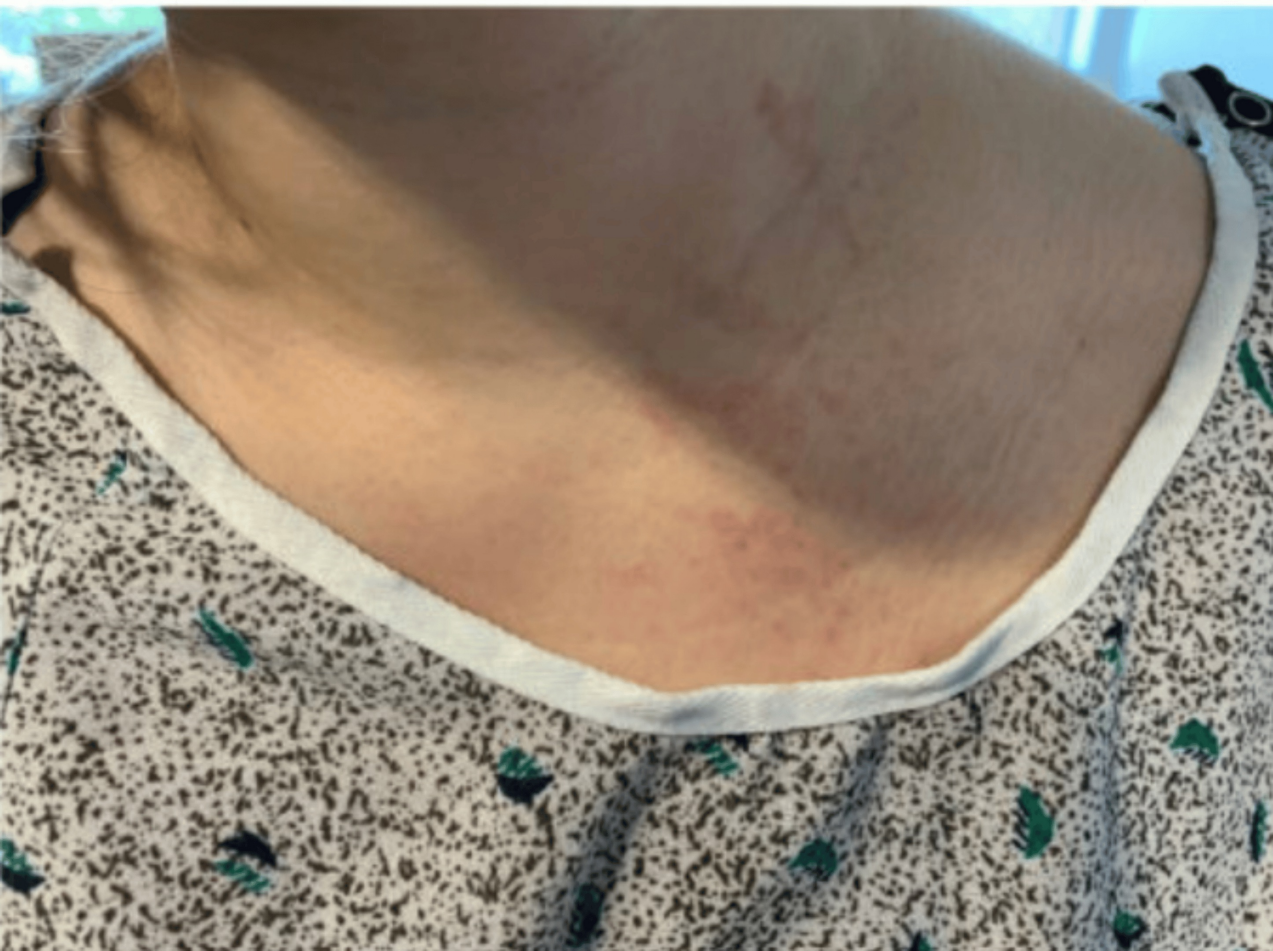
Salmon-colored maculopapular rash around the neck

A 24-year-old female with no known past medical history was admitted to the inpatient unit for the management of a fever of unknown origin. The patient first developed a sporadic, erythematous, nonpruritic maculopapular rash during a trip to Mexico two months before she was admitted to the hospital (Figures 1, 2). She considered them “hives” and took cetirizine, which temporarily resolved the rash. However, the rash reappeared once she was back in the United States. This time, the rash worsened and spread over her entire body.

In addition to the rash, she also had a fever and symptoms of pharyngitis, along with lymphadenopathy, at the same time the rash appeared. The patient also began to experience diffuse migratory arthralgias, starting in her left fingers and wrist and moving to her right ankle and left knee. She initially presented to an urgent care, where she received an intramuscular corticosteroid injection along with a tapering dose of steroids, which provided temporary relief. However, she relapsed immediately after completing the steroid course. She then worsened clinically, with the rash spreading to her trunk, back, and all four extremities, along with significant left knee pain. She subsequently developed pharyngitis with lymphadenopathy and a fever of 39.3°C, prompting evaluation in the emergency department.

She was tachycardic and febrile on presentation. Initial workup was significant for leukocytosis, elevated inflammatory markers, normal rheumatoid factor, elevated liver enzymes, and a ferritin level of 29,972 ng/mL (Table 1). After an infectious workup, including bacteriological, viral, and parasitic panels, as well as blood cultures and urinalysis, was initiated and preliminary results came back negative, the most likely diagnosis was determined to be adult-onset Still’s disease.

**Table 1:** Initial laboratory test results ANA: antinuclear antibody, ANCA: antineutrophil cytoplasmic antibody, ALT: alanine aminotransferase, AST: aspartate aminotransferase, CRP: C-reactive protein, ESR: erythrocyte sedimentation rate, ISR: immune status ratio, LDH: lactate dehydrogenase, RF: rheumatoid factor.

Test	Value	Reference range
Total leukocyte count	15,700/µL	3400-10,500/µL
Neutrophils	81.3%	45-75%
CRP	20.8 mg/dL	<0.9 mg/dL
ESR	57 mm/hour	<20 mm/hour
RF titer	13 IU/mL	0-13 IU/mL
Ferritin	29,972 ng/mL	20-324 ng/mL
Troponin	118 ng/L	0-10 ng/L
D-dimer	11.19 mg/l	<0.49 mg/l
N terminal pro-B- type natriuretic peptide	464 pg/mL	0-125 pg/mL
Albumin	2.6 g/dL	3.4-5 g/dL
AST	407 U/L	10-35 U/L
ALT	316 U/L	10-35 U/L
LDH	913 U/L	107-230 U/L
ANA screen	0.43 ISR	<0.99 ISR
ANCA panel (anti-myeloperoxidase and proteinase-3)	Negative	N/A

During the hospital stay, the patient's liver enzymes and cell counts continued to increase. A CT scan of the neck showed nonspecific, mildly prominent precarinal lymph nodes, while a CT scan of the abdomen and pelvis revealed a mildly enlarged liver and spleen, raising concern for macrophage activation syndrome. The patient was immediately started on high-dose intravenous steroids, which led to improvement in cell counts and overall symptoms. With clinical improvement, she was discharged on a tapering dose of oral steroids and instructed to follow up with the rheumatology outpatient clinic. Since then, she has been following up with rheumatology and remains in remission.

## Discussion

AOSD is an inflammatory disorder characterized by quotidian fevers, arthralgias, a salmon-colored maculopapular rash, and an elevated white blood cell count [1,6]. AOSD is a diagnosis of exclusion, and in most cases, the typical features mentioned earlier are absent [7]. Although elevated ferritin is not pathognomonic, it may help diagnose the disease [1,6]. Hyperferritinemia is most helpful when present in combination with other clinical signs and symptoms of AOSD. The ferritin level in patients with AOSD typically exceeds five times the normal value and is usually > 1,000 ng/dL, which is associated with high sensitivity for the disease [1,8]. Testing for both total serum ferritin and the glycosylated fraction yields higher specificity than either test alone [9]. Due to the lack of definitive diagnostic tests, several diagnostic criteria have been proposed for the diagnosis of AOSD, among which Yamaguchi’s criteria, introduced in 1992, and the 2002 Fautrel’s criteria are the most commonly used [10,11].

The diagnosis of AOSD can be challenging, as patients may present with a variety of nonspecific symptoms. Maintaining a high clinical suspicion for AOSD is important to prevent delays in treatment and complications. One feared and potentially fatal complication of AOSD is MAS. MAS is a reactive form of hemophagocytic lymphohistiocytosis (HLH) that occurs in the setting of systemic rheumatic diseases [12]. It is a rare manifestation and can develop as a complication of Still’s disease in both children and adults. A study using the National Inpatient Sample database showed that 1.7% of patients with AOSD developed MAS [13]. Studies have shown that MAS and disseminated intravascular coagulation (DIC) are the leading causes of death in patients with AOSD [13,14]. Thus, recognizing MAS in patients with AOSD is very important [14].

Concern for MAS is particularly high in patients with ferritin levels elevated out of proportion to other inflammatory markers, lymphadenopathy, weight loss, transaminase elevation, hepatomegaly, marked elevation in D-dimer, thrombocytopenia, and/or a decreasing erythrocyte sedimentation rate despite continued elevation of C-reactive protein [14]. Notable liver dysfunction and low neutrophils are both poor prognostic factors in MAS, as are low platelets, low fibrinogen, and high ferritin levels [14]. Awareness of this complication is crucial, as the mortality rate in AOSD-associated MAS is 10-20% [14,15]. Bone marrow examination demonstrating well-differentiated macrophages (histiocytes) actively phagocytosing hematopoietic elements is the hallmark of MAS diagnosis [16].

Management of new-onset AOSD depends on disease severity. For patients with mild disease (i.e., rash, fever, mild arthralgia, and no MAS), nonsteroidal anti-inflammatory drugs (NSAIDs) are recommended [17]. Similarly, patients with moderate to severe disease who are resistant to NSAID monotherapy are treated with anakinra, glucocorticoids, or other DMARDs [18]. However, if there is concern for MAS, combination therapy with anakinra and glucocorticoids is recommended [19]. Prompt diagnosis and early treatment significantly improve morbidity and mortality.

## Conclusions

In summary, clinicians need to be able to identify AOSD in its early stages to prevent patients from developing the lethal complication of macrophage activation syndrome. Early diagnosis is challenging due to the heterogeneous symptoms and multisystem involvement of the disease. However, careful evaluation and appropriate implementation of diagnostic tests can help identify the disease in a timely manner and initiate treatment. Even if a patient develops MAS, starting the right treatment regimen can reduce inflammation, and the patient may achieve remission without long-term disability from the disease.
